# A Rare Case of Supinator Intramuscular Myxoma with Encasement of the Posterior Interosseous Nerve

**DOI:** 10.1155/2019/5156032

**Published:** 2019-08-14

**Authors:** Chiao Yee Lim, Suryasmi Duski, Ping Ching Chye

**Affiliations:** Department of Orthopaedic Surgery, Kuala Lumpur Hospital, 50586 Kuala Lumpur, Malaysia

## Abstract

Intramuscular myxomas are rare, benign mesenchymal tumors. Tumor location in the forearm is very rare among all the intramuscular myxomas. To the best of our knowledge, there were two cases of supinator intramuscular myxoma reported in the literature, and we intend to report the third case of supinator myxoma with encasement of the posterior interosseous nerve (PIN). A 67-year-old lady presented with history of left proximal forearm swelling for the past 5 years. Magnetic resonance imaging showed a lobulated multiseptated lesion seen within left supinator muscle, which was hyperintense on T2-weighted images (T2WI) and hypointense on T1-weighted images (T1WI), with peripheral enhancement post contrast. The tissue diagnosis of myxoma was confirmed via an open biopsy. She underwent en bloc resection of the tumor. The PIN was encased by the tumor; it was preserved and carefully released from the tumor. The nerve sheath served as an excision margin. In conclusion, we present a rare case of an intramuscular myxoma in the supinator muscle. In view of the location, extra attention should be paid during excision surgery to locate the PIN and to avoid damaging the surrounding structures.

## 1. Introduction

Intramuscular myxomas are rare, benign mesenchymal tumors in the musculoskeletal system, in which the primitive mesenchymal cells lose their capacity to produce collagen but instead have excess hyaluronic acid and immature collagen production [1]. Tumor location in the forearm is very rare among all the intramuscular myxomas. To the best of our knowledge, there were two cases of supinator intramuscular myxoma reported in the English literature [2, 3], and we intend to report the third case of supinator myxoma with encasement of the posterior interosseous nerve (PIN).

## 2. Case Report

A 67-year-old lady, with underlying type 2 diabetes mellitus, presented with the history of left proximal forearm swelling which had progressively increased in size for the past 5 years. There was no pain and no numbness or weakness of her left wrist or fingers. She denied any past history of trauma, infection, fever, or constitutional symptoms. Physical examination revealed an ill-defined swelling at the radial border of the left proximal forearm. It was nontender, was not attached to overlying skin but fixed to the underlying structures, had a smooth surface, and was firm to hard in consistency.

**Figure 1:**
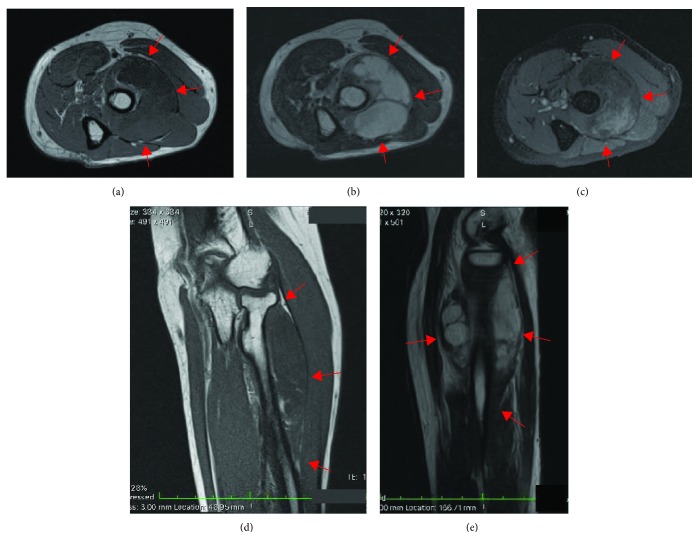
Magnetic resonance imaging (MRI) showed a lobulated multiseptated lesion seen within left supinator muscle as delineated by the red arrows, which showed a hyperintense signal on T2-weighted images (T2WI) (b, e) and hypointense on T1-weighted images (T1WI) (a, d), with peripheral enhancement post contrast (c).

Plain radius and ulna radiographs did not show any scalloping, osteolysis, pathological fracture, or soft tissue calcification. Magnetic resonance imaging (MRI) showed a lobulated multiseptated lesion seen within left supinator muscle, which showed hyperintense signal on T2-weighted images (T2WI) and hypointense on T1-weighted images (T1WI), with peripheral enhancement post contrast (Figures 1(a)–1(e)). The PIN was reported as being encased/impinged by the tumor. An open biopsy was performed, and histopathological examination confirmed the diagnosis of myxoma.

**Figure 2:**
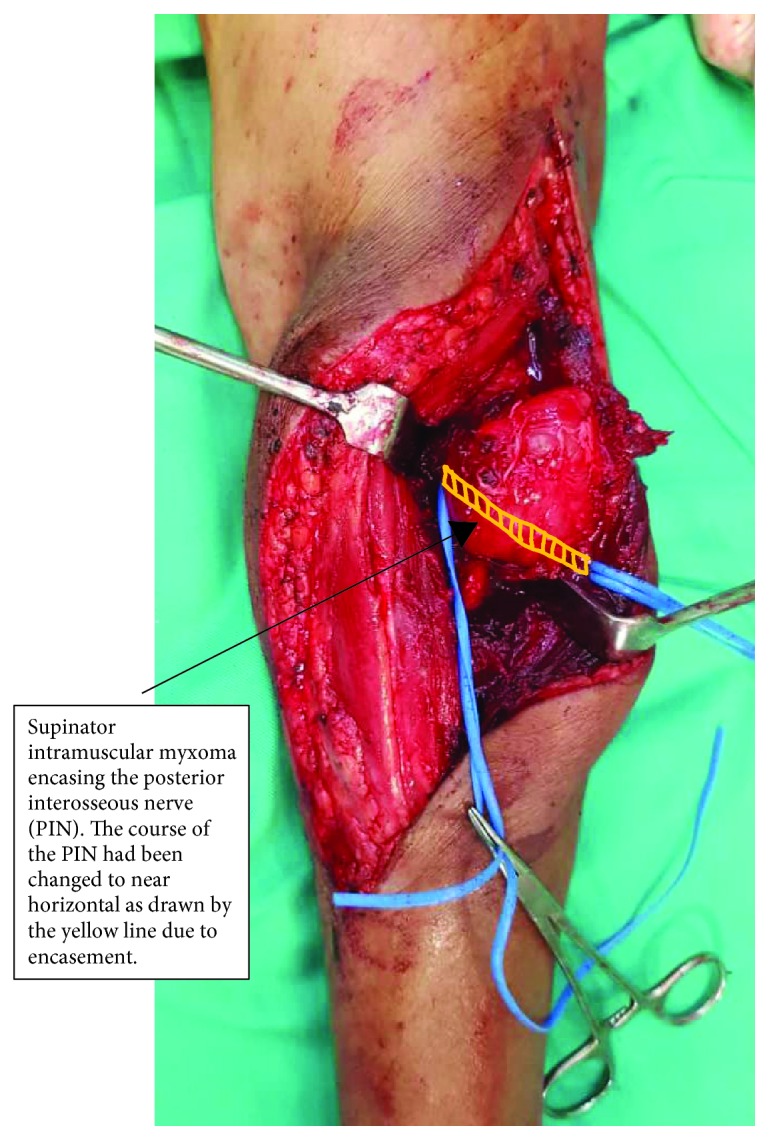
Intraoperative photograph showed the left supinator intramuscular myxoma encasing the posterior interosseous nerve (PIN). The course of the PIN had been changed to near horizontal as drawn by the yellow line due to encasement.

She was counselled for tumor excision with possible risk of postoperative loss of PIN function informed. The patient was fully aware about the possibility of PIN injury and consented for the surgery. The patient underwent en bloc resection of her left supinator intramuscular myxoma under general anesthesia. A curvilinear longitudinal skin incision was made overlying the tumor mass. A surgical plane was attained between the extensor carpi radialis brevis and extensor digitorum communis to reach the tumor mass. The tumor was noted to be a well-encapsulated cystic, solid, well-defined supinator intramuscular mass which encroached the proximal radius. The PIN was encased by the tumor (Figure 2). The PIN was carefully released from the tumor under loupe magnification. The excised tumor measured at 5.5 × 4.5 × 2.5 cm^3^. Clinical examination postoperatively showed no wrist or finger drop. Her recovery was uneventful. Final histopathological examination of the excised tumor confirmed the diagnosis of intramuscular myxoma with hypocellular and loosely arranged spindle to stellate cells set within a copious myxoid stroma. The cells appeared bland looking and displayed coarse chromatin, inconspicuous nucleoli, and scanty cytoplasm. No mitotic activity was observed. At the time of this report, the patient is currently well at 6 months post surgery; we will continue to follow up on her with 6-monthly appointment.

## 3. Discussion

Intramuscular myxoma was first delineated as a definite clinicopathologic lesion by Enzinger in 1965 [4]. The incidence of intramuscular myxoma varies between 0.10 and 0.13 per 100,000 [2, 5]. The majority of lesions present in the fourth to sixth decade of life with slight female predominance [2, 5]. It typically involves large muscle groups in the thigh, gluteal region, shoulder, and upper arm [6]. Intramuscular myxoma occurs as a solitary entity, less frequently in association with fibrous dysplasia of the bone (Mazabraud's syndrome), or as a part of the McCune-Albright syndrome (polyostotic fibrous dysplasia, café-au-lait spots, and endocrine hyperfunction) [7]. In these syndromes, multiple myxomas are encountered more often [7].

On T2-weighted MRI, a perilesional fat rind and increased signal intensity in the adjacent muscle tissue may be observed in intramuscular myxoma [6]. On T1-weighted MRI, the tumor has a low signal intensity [6]. On contrast-enhanced MRIs, myxomas usually demonstrate a mild to moderate contrast enhancement. Cystic areas may be observed in >50% of all lesion with a thin peripheral and septal enhancement pattern [6, 8–11]. However, intramuscular myxoma has nonspecific clinical symptoms and radiologic findings that may be confused with other soft tissue tumors; microscopic examination is required to confirm the diagnosis.

To the best of our knowledge, this is the third case of supinator intramuscular myxoma reported in the English literature. The first case was reported by Kursumovic et al. [2]; the patient presented with PIN palsy after bowel surgery associated with intramuscular myxoma of the supinator muscle. Their patient underwent tumor excision 3 months after the symptom onset, and intraoperatively, the PIN was found alongside of the tumor and was compressed and flattened against the distal rim of the supinator muscle [2]. Nonaka et al. reported the second case of intramuscular myxoma in the supinator muscle with transient postoperative PIN palsy due to soft tissue retraction during the excision surgery [3]. Their patient had complete PIN function recovery at 3 months after the surgery [3].

Our patient had a relatively large supinator intramuscular tumor (the excised mass measured at 5.5 × 4.5 × 2.5 cm^3^) which encased the PIN. The preoperative biopsy had confirmed the benign nature of the tumor; thus, during the surgery, the PIN was preserved and carefully released from the tumor. The nerve sheath served as an excision margin.

In conclusion, we described a rare case of an intramuscular myxoma in the supinator muscle. The treatment of the tumor is en bloc excision. However, in view of the location, extra attention should be paid to locate the PIN and to avoid damaging the surrounding structures. Recurrence after intramuscular myxoma excision is uncommon [12]; thus, we should avoid damaging any important neurovascular structures by overzealous attempt to remove all tumor tissues.
